# A Flexible Sensing Unit Manufacturing Method of Electrochemical Seismic Sensor

**DOI:** 10.3390/s18041165

**Published:** 2018-04-11

**Authors:** Guanglei Li, Zhenyuan Sun, Junbo Wang, Deyong Chen, Jian Chen, Lianhong Chen, Chao Xu, Wenjie Qi, Yu Zheng

**Affiliations:** 1 University of Chinese Academy of Sciences, Beijing 100049, China; liguanglei13@mails.ucas.edu.cn (G.L.); dychen@mail.ie.ac.cn (D.C.); chenjian@mail.ie.ac.cn (J.C.); chenlianhong15@mails.ucas.edu.cn (L.C.); xuchao16@mails.ucas.edu.cn (C.X.); qiwenjie16@mails.ucas.edu.cn (W.Q.); 2 Institute of Electronics, Chinese Academy of Sciences, Beijing 100010, China; 3 Tsinghua University, Beijing 100084, China; sunzhenyuan@mail.tsinghua.edu.cn; 4 Affiliated High School of Peking University, Beijing 100080, China; zhengyu@stu.pkuschool.edu.cn

**Keywords:** electrochemical seismic sensor, parylene substrate, sensitivity increasing, noise level

## Abstract

This paper presents an electrochemical seismic sensor in which paraylene was used as a substrate and insulating layer of micro-fabricated electrodes, enabling the detection of seismic signals with enhanced sensitivities in comparison to silicon-based counterparts. Based on microfabrication, paralene-based electrochemical seismic sensors were fabricated in which the thickness of the insulating spacer was 6.7 μm. Compared to silicon-based counterparts with ~100 μm insulating layers, the parylene-based devices produced higher sensitivities of 490.3 ± 6.1 V/(m/s) vs. 192.2 ± 1.9 V/(m/s) at 0.1 Hz, 4764.4 ± 18 V/(m/s) vs. 318.9 ± 6.5 V/(m/s) at 1 Hz, and 4128.1 ± 38.3 V/(m/s) vs. 254.5 ± 4.2 V/(m/s) at 10 Hz. In addition, the outputs of the parylene vs. silicon devices in response to two transit inputs were compared, producing peak responses of 2.97 V vs. 0.22 V and 2.41 V vs. 0.19 V, respectively. Furthermore, the self-noises of parylene vs. silicon-based devices were compared as follows: −82.3 ± 3.9 dB vs. −90.4 ± 9.4 dB at 0.1 Hz, −75.7 ± 7.3 dB vs. −98.2 ± 9.9 dB at 1 Hz, and −62.4 ± 7.7 dB vs. −91.1 ± 8.1 dB at 10 Hz. The developed parylene-based electrochemical seismic sensors may function as an enabling technique for further detection of seismic motions in various applications.

## 1. Introduction

A seismic sensor is a key sensing element widely used in the fields of geophysical exploration and seismic monitoring [1,2,3,4,5]. There are a variety of commercialized seismic sensors such as moving-coil seismic sensors [6,7,8], optical-fiber seismic sensors [9,10], capacitive seismic sensors [11,12], MEMS (Micro-Electro-Mechanical System) seismic sensors [13,14,15], and electrochemical seismic sensors [16,17,18,19,20,21,22,23,24,25,26,27,28,29,30,31]. The electrochemical seismic sensors are under extensive research, and they feature lower self-noises, higher performance in the low-frequency domain, and wider installation angels than the other kinds of seismic sensors due to the liquid inertial mass [17].

The “solion”, which was the prototype of the electrochemical seismic sensors, was initially presented in the 1950s by US-Navy [19,20]. Early applications of “solion” devices included the detection of low-frequency acoustic waves, either in the form of infrasonic microphones or limited-band seismometers [19,20,21,22]. Significant improvements for motion detections were further conducted in Russia, in which the concept of the electrochemical seismic sensor was introduced [23,24].

However, the conventional electrochemical seismic sensors were fabricated with traditional net-waving technology [24], which resulted in high manufacturing costs and a low alignment success rate, thus resulting in poor consistencies. The prototype of the electrochemical seismic sensors based on MEMS technologies was successfully manufactured by He et al. in 2012 [26,27], in which the inclusion of microfabrication technologies can realize quantitative productions of electrochemical seismic sensors, thus reducing fabrication costs. However, the problem of low consistency was not addressed until the proposed integrated electrodes by Deng et al. in 2014 [17], in which the fabrication of the integrated electrodes got rid of the step of alignments, enhancing the consistency of the seismic sensors. However, in these micro-fabricated electrochemical seismometers, the sensing electrodes were all fabricated on silicon wafers, with the thickness of the insulating layers in the range of 100 μm, leading to compromised sensitivities. To address this issue, this study proposed a new method to fabricate the sensing units based on parylene (a more flexible material than the conventional silicon) rather than silicon, thus decreasing the thickness of the insulating layers from hundreds of micrometers to several micrometers with a more than 10-fold increase in sensitivities.

The structure of this paper is organized as follows. Section 2 introduces the working principle of the electrochemical seismic sensor and shows how the thickness of the insulating spacers affected the performances of the electrochemical seismic sensors theoretically. Section 3 introduces the results of the numerical simulations. Section 4 illustrates the fabrication processes and the assembling of the electrochemical seismic sensor. Section 5 introduces the characteristics of the proposed electrochemical seismic sensors based on flexible sensing units.

## 2. Structure and Working Principle

**Figure 1:**
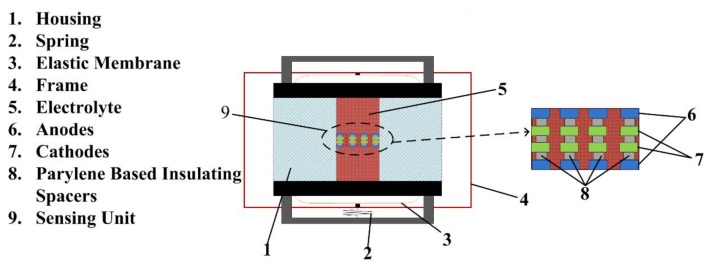
The schematic of the parylene-based MEMS electrochemical seismic sensor is composed of a sensing unit including porous sensing electrodes immersed in an electrolyte solution sealed in a plexiglass house by elastic membranes. In response to external seismic signals, the electrolyte moves in an opposite direction to the external vibration, leading to ion concentration gradients and raw current outputs between the anode-cathode pairs. The sensitivity of the micro-fabricated electrochemical seismic sensors was enhanced due to the decreasing of the insulating spacers, which was realized by using the parylene substrates instead of the silicon substrates.

The sensing unit, which includes two anodes, two cathodes, and parylene-based insulating spacers between the electrodes, is the key component of the electrochemical seismic sensor (shown in Figure 1). The electrodes and the insulating spacers based on parylene are fabricated with through holes, and thus the electrolytes (the mixed solution of I_2_ and KI in which the concentration of the KI was 50–100 times of the concentration of the I_2_) can pass through. The electrodes and electrolytes were sealed in a plexiglass housing.

When a DC voltage (0.2–0.4 V) is applied to the electrodes, there are reversible electrochemical reactions at the electrodes as follows:

(1) $$3I^{-}\overset{2e^{-}}{\Leftrightarrow }I_{3}^{-}$$

A given acceleration on the seismic sensor causes the relative motion between the electrodes and the electrolytes, which results in gradient concentration variations of the electrolytes on the surfaces of the electrodes, generating output currents detected by the detecting circuit. The output current can be further processed to be either in proportion to the input velocity or the input acceleration. The electrical currents through the electrodes can be related to the following equation:

(2) $$I=qF\left(\oint_{S}^{}\left(J,n\right)\mathrm{dS}\right)$$

in which *q* = 1 is the charge transferred across the interface in an electrochemical reaction, *F* = 9.65 ×10^5^ is Faraday constant, J is the flux of the ions on the surface of the electrodes ($I^{-}$ at the anodes and $I_{3}^{-}$ at the cathodes), *n* is a unit vector normal to the surface of the electrode, and S is the electrode surface area. Therefore, the differential current output of two cathodes is given by:

(3) $$I_{out}=qF\left[\oint_{S_{c2}}^{}\left(J,n\right){\mathrm{dS}}_{c2}-\oint_{S_{c1}}^{}\left(J,n\right){\mathrm{dS}}_{c1}\right]$$

in which $S_{c1}$ and $S_{c2}$ represent the areas of the two cathodes, respectively. J is the flux of $I_{3}^{-}$ on the surfaces of the two electrodes. Since there are a lot of inert ions such as *K^+^*, the flux of the ions can be expressed by simplifying the Nernst-Planck equations:

(4) J=−D∇C+CV

The flux of the ions on the surfaces of the electrodes ($I^{-}$ at the anodes and $I_{3}^{-}$ at the cathodes) follows the Butler-Volmer equation according to the rate-determining step (1):

(5) $$2\left(J_{I_{3}^{-}},n\right)=-\frac{2\left(J_{I^{-}},n\right)}{3}\;\;=-KC_{I^{-}}\exp\left[\frac{\left(1-\alpha \right)qF}{RT}*\left(U-\varphi -E^{0}\right)\right]-2KC_{I_{3}^{-}}\exp\left[-\frac{\alpha qF}{RT}*\left(U-\varphi -E^{0}\right)\right]$$

in which D is the diffusion coefficient and *C* is the concentration of the ions ($I^{-}$ at the anodes and $I_{3}^{-}$ at the cathodes), *V* is the velocity of the electrolyte, *K* is the constant of the electrode reaction velocity, U is the electrical potential between the electrodes, φ is the electrical potential of the electrolyte, $E^{0}$ is the equilibrium electrical potential, and α is the transfer coefficient of the reaction on the cathodes. In addition, the motion of the electrolyte follows the Navier-Stocks equation:

(6) $$\frac{\partial V}{\partial t}=-\left(V*\nabla \right)*V-\frac{\nabla P}{\rho }+\frac{\mu }{\rho }\nabla^{2}V+g$$

in which *P* is the pressure, g is acceleration of gravity, and ρ and μ are the density and viscosity of the electrolyte. According to (3)–(6), $I_{out}$ can be translated from the concentration of $I_{3}^{-}$.

## 3. Numerical Simulation

**Figure 2:**
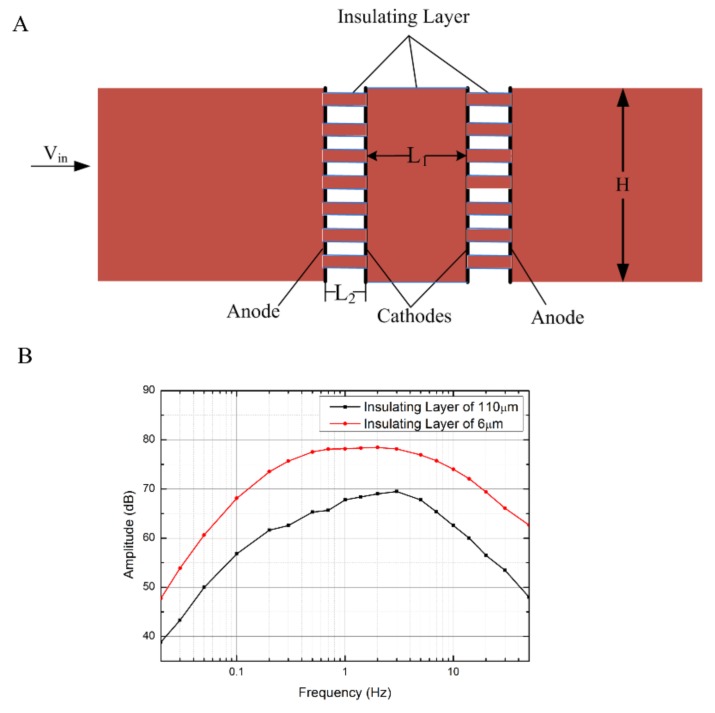
(**A**) Two-dimensional simulation model of the electrochemical seismic sensor using COMSOL Multiphysics. L_1_ represents the insulating spacer between the two cathodes, L_2_ represents the insulating spacer between the cathode and the anode, H represents the height of the flow path, and V_in_ represents the speed of the electrolyte. (**B**) The simulation output of the electrochemical seismic sensors with two insulating spacers with thicknesses of 110 μm and 6 μm, respectively, demonstrates that the output sensitivities of the electrochemical seismic sensors were inversely correlated with the thickness of the insulating spacers between the cathodes and the anodes.

Finite element simulations are effective methods in accurate parameter design and optimization, since the general solution is difficult to obtain according to (4)–(6). Figure 2A presents the simulation model (COMSOL Multiphysics 3.5, Stockholm, Sweden) of the electrochemical seismic sensor, in which four electrodes were arranged in an anode-cathode-cathode-anode sequence with insulating spacers between. A set of geometrical parameters is employed as follows: L_1_ of 130 μm represents the insulating spacer between the two cathodes; H of 500 μm represents the height of the flow path; L_2_ represents the thickness of the insulating layer between the anode and the cathode; and *V_in_* represents the speed of the electrolyte, which is presented at the entrance of the flow path. The input velocity of the simulation model was defined as follows:

(7) $$V_{in}\left(t\right)=\{\begin{array}{c} 0\;t<2000\;s\\ v_{p}\sin\left(2\pi ft\right)\;{\mathrm{ms}}^{-1}\;t\geq 2000\;s\; \end{array}$$

in which $v_{p}=10^{-5}{\mathrm{ms}}^{-1}$ and *f* is the frequency of the input signal, ranging from 0.02 Hz to 50 Hz, while the input signal is zero as a stable time of the electrochemical system. In addition, the initial concentration of the I_2_ and KI is 0.04 mol/L and 4 mol/L, respectively.

The coupling fields in this simulation model include laminar flow field and electrochemical analysis field, which contain Navier–Stokes incompressible fluid equations, Nernst–Planck ion transport equation, and Butler–Volmer electrode boundary condition, in which the boundary conditions on electrodes were given as follows: q = 1, *K* = 6.8 × 10^−5^ m/s, *E*^0^ = 0.54 V, α = 0.5, the diffusion coefficient of $I_{3}^{-}$ = 0.55 × 10^−9^ m^2^/s, and the diffusion coefficient of $I^{-}$ = 1.22 × 10^−9^ m^2^/s.

The transient solver was used to simulate the model. It is noted that in order to ensure that there are sufficient data points in one cycle, the step length should be smaller when the input frequency is higher. Then, the cathodes outputs were obtained by the line integral of the local current density on the two cathodes.

Figure 2B illustrates the simulation results of the electrochemical seismic sensors with two different thicknesses of insulating spacers between cathodes and anodes, which were 110 μm and 6 μm, respectively. The simulation results demonstrated that the output sensitivity of the electrochemical seismic sensor in the frequency domain of 0.02–50 Hz was inversely correlated with the thickness of the insulating spacer between the cathode and the anode. The silulation results provided the basis for parameter optimization of the sensing unit of the electrochemical seismic sensor.

## 4. Fabrication and Assembling

**Figure 3:**
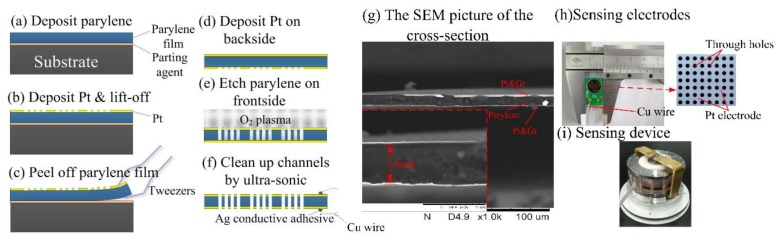
(**a**–**f**) The fabrication process of the sensing electrodes. (**a**) The parylene was deposited on a reusable substrate. (**b**) The porous platinum (Pt) electrodes were formed on the parylene layer using the lift-off technology. (**c**) The parylene film was peeled away from the substrate. (**d**) Pt sputtering on the other side of the parylene film. (**e**) The parylene film was etched through by oxygen plasma from the front side. (**f**) The suspended Pt electrodes with the beneath parylene etched away were removed by the ultra-sonic cleaning. (**g**) SEM picture of the cross section of the fabricated electrodes. The thickness of the parylene based insulating layer was quantified as 6.7 μm. (**h**−**i**) The flexible electrodes and the assembled electrochemical seismic sensor.

Figure 3a–f shows the fabrication process of the sensing units in which parylene was used as the substrate for electrode patterning and at the same time as the insulating layer:

(a) The parylene was deposited on a reusable substrate. The substrate used could be a glass wafer or a silicon wafer. The thickness of parylene film was well regulated by the mass of the raw materials (parylene) used. Note that the substrate should be cleaned up with concentrated sulfuric acid and deionized water sequentially to remove organic impurities and mental ions. In addition, the substrate should be polished by release agent to ensure the parylene film is more easily peeled away from the substrate.

(b) The chrome (Cr) (20 nm in thickness) and the platinum (Pt) (200 nm in thickness) was sputtered on the surface of the parylene film with photoresist mask sequentially. To increase the adhesive force between the parylene film and the Pt ions, the cleaning of oxygen ion etching (3 min) is needed before sputtering. Then, the porous Pt electrodes were formed on the parylene layer after the film was washed ultrasonically in acetone, ethyl alcohol, and deionized water sequentially.

(c) The parylene film was peeled away from the substrate by flat tweezers. Care should be taken with the process in case the Pt electrodes fall off the parylene film.

(d) After the cleaning of oxygen ion etching (3 min), the chrome (Cr) (20 nm in thickness) and the Pt (200 nm in thickness) sputtering on the other side of the parylene film directly without photoresist was masked sequentially.

(e) The parylene film was etched through by oxygen plasma from the front side with the porous Pt electrodes as the self-aligned etching mask. About 40 min were needed to etch through the parylene film.

(f) The Pt electrodes suspended on the electrode holes were removed by the ultra-sonic cleaning in deionized water. Then, the parylene based electrodes were left to dry in an oven at 80 °C. At last, the wires were connected to the electrodes.

Figure 3g shows an SEM (scanning electron microscope) picture of the cross section of the fabricated electrodes in which parylene was used as the electrode substrate and the insulating layer. The thickness of the parylene-based insulating layer was quantified as 6.7 μm. Figure 3h,i shows the manufactured flexible electrodes and the assembled electrochemical seismic sensor. The electrodes of the electrochemical seismic sensor were packaged based on mechanical compression. Note that polymeric glues should be avoided in the packaging step in case the glues can absorb solutes of the electrolyte solution, leading to a gradual decrease of the sensitivity of the seismic sensor as time goes by.

Note that since the parylene-based electrodes were ultra-thin (6.7 μm) and fragile, the wires connected to the electrodes should not be too stiff. Thus, the cooper filaments were used as the connecting wires in this study. Moreover, the Ag conductive adhesive instead of the soldering process was used to bond the cooper wires to the electrodes, and thus the concern of parylene damage and short circuits of the two electrodes was properly addressed.

## 5. Devices Characterizations

### 5.1. Sensitivity Characterization

**Figure 4:**
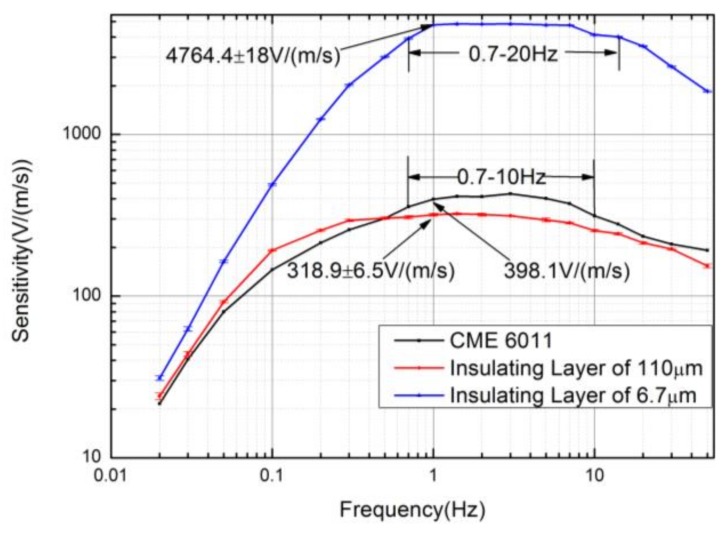
The sensitivities of the electrochemical seismic sensors that relied on parylene substrates and silicon substrates, as well as commercial seismic sensor CME6011. The insulating layer made of parylene was quantified as 6.7 μm in thickness, and that made of silicon wafer was 110 μm in thickness. The ultra-thin insulating layers based on parylene were observed to produce a significant increase in device sensitivity.

The experiments of measuring sensitivity characteristics were conducted on an ultra-low-frequency vibration table (National Institute of Metrology). Figure 4 shows the sensitivities of the electrochemical seismic sensors relying on parylene substrates with the thickness of the insulating spacer as 6.7 μm and the silicon substrates with the thickness of the insulating spacer as 110 μm, as well as the commercial seismic sensor CME6011. The seismic sensor that relied on parylene and silicon substrate produced results of (490.3 ± 6.1 V/(m/s) vs. 192.2 ± 1.9 V/(m/s)) at 0.1 Hz. (4764.4 ± 18 V/(m/s) vs. 318.9 ± 6.5 V/(m/s)) at 1 Hz. (4128.1 ± 38.3 V/(m/s) vs. 254.5 ± 4.2 V/(m/s)) at 10 Hz. The sensitivities of the latter were comparable with those of CME6011.

The thickness of the insulating layer was decreased about 16.4 times from 110 μm to 6.7 μm, and the sensitivities of the device were increased 2.55 times (8.1 dB) at 0.1 Hz, 14.9 times (23.4 dB) at 1 Hz, and 16.2 times (24.2 dB) at 10 Hz, producing results consistent with theoretical analysis.

Note that the 3 dB (the frequency range that the amplitude is the 0.707 times the maximum) working bandwidth of the parylene-based devices was lower than the silicon-based counterparts. However, the bandwidth of the parylene-based devices was comparable to the commercial seismic sensor CME6011 (0.7–20 Hz vs. 0.7–10 Hz). The tradeoff between the sensitivity and the working bandwidth should be well balanced according to the requirements of the specific applications.

### 5.2. Transient Response

**Figure 5:**
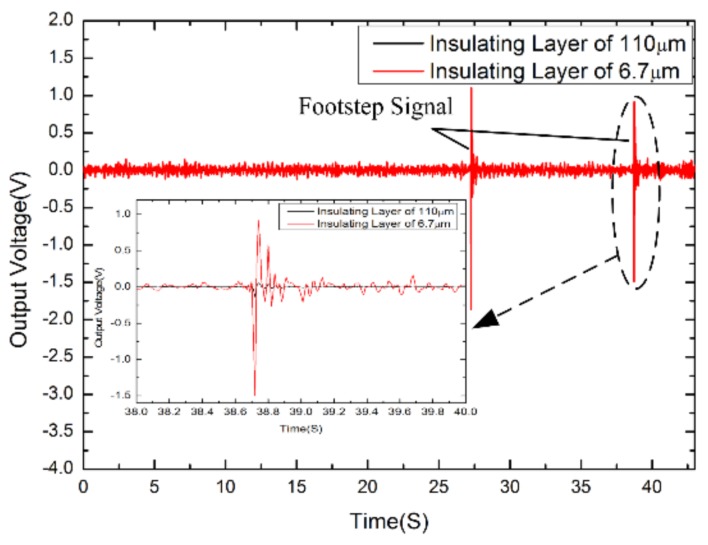
The inputs of footsteps were detected by the parylene vs. silicon based electrochemical seismic sensors, respectively. The source of footsteps was 2 m away from the devices. The peak responses of the parylene based seismic sensors were about 13.3 times (27.26–27.28 s) and 12.5 times (38.72–38.74 s) more than the seismic sensors based on the silicon substrates in response to two inputs of footsteps.

Figure 5 shows the transient responses of the electrochemical seismic sensors based on the parylene substrate and the silicon substrate, respectively. After the seismic sensors worked in a stable stage in a quiet environment, the input signals of footsteps were applied by the experimenter two meters away from the sensors.

As shown in Figure 5, the footsteps were applied at 27.26–27.28 s and 38.72–38.74 s. The responses of these two types of sensors were observed 2.97 V vs. 0.22 V (27.26–27.28 s) and 2.41 V vs. 0.19 V (38.72–38.74 s), which demonstrated that the transient responses of the seismic sensor based on the parylene substrate were about 13.3 times and 12.5 times those of the seismic sensor based on the silicon substrate, respectively.

The results validated the notion that the instantaneous sensitivity increased with the decreasing of the thickness of the insulating spacer.

### 5.3. Self-Noise Levels

Self-noise is a very important parameter of a seismic sensor. The seismic sensors based on parylene substrates, silicon substrates, and commercial seismic sensor CME6011 were placed side-by-side in a basement with reduced environmental interferences. The self-noise data was collected when the seismic sensors worked in a stable state.

**Figure 6:**
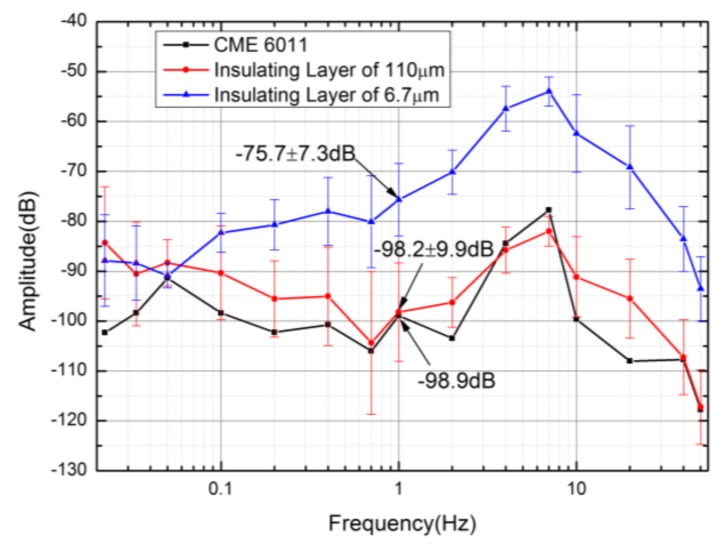
The self-noise power spectrums of electrochemical seismic sensors that relied on parylene and silicon substrates, respectively, as well as commercial seismic sensor CME6011. The self-noise levels of the seismic sensors based on the parylene substrate were about 82.3 ± 3.8 dB@ 0.1 Hz, 75.7 ± 7.3 dB@ 1 Hz, and 62.4 ± 7.7 dB@ 10 Hz, which were higher than those of the seismic sensors based on the silicon substrates.

The self-noise power spectrum density of the three seismic sensors was shown in Figure 6. The self-noise power spectrum density was quantified according to the output voltages of the seismic sensors without inputs (0 dB represents 1 V$/\sqrt{\mathrm{Hz}}$). The self-noises of the seismic sensors relying on parylene and silicon substrate were −82.3 ± 3.9 dB vs. −90.4 ± 9.4 dB at 0.1 Hz, −75.7 ± 7.3 dB vs. −98.2 ± 9.9 dB at 1 Hz，and −62.4 ± 7.7 dB vs. −91.1 ± 8.1 dB at 10 Hz, which demonstrated that the self-noises of the seismic sensor based on the parylene substrate were about 8.1 dB, 22.5 dB, and 28.7 dB higher than those of the seismic sensor based on the silicon substrate, which were almost equal to the sensitivity ratios of the two kind of seismic sensors (8.1 dB at 0.1 Hz, 23.4 dB at 1 Hz, and 24.2 dB at 10 Hz). This meant that the noise levels of the two kinds of electrochemical seismic sensors were comparable when the sensitivity was normalized. In addition, the self-noise of the seismic sensor based on the parylene substrate was comparable to that of CME6011 at 1 Hz when the sensitivity was normalized.

## 6. Conclusions

This paper demonstrated an electrochemical seismic sensor in which paraylene was used as a substrate and an insulating layer of micro-fabricated electrodes, enabling the detection of seismic signals with enhanced sensitivities in comparison to silicon-based counterparts. Paralene-based electrochemical seismic sensors were fabricated using micro-fabrication technologies with a thickness of 6.7 μm as the insulating layers. Compared to the silicon-based counterparts, the parylene-based devices produced higher sensitivities and comparable noise levels when the sensitivity was normalized. Thus, the developed electrochemical seismic sensors based on parylene may function as an enabling technique for further detections of seismic motions in various applications.
